# Painless Effusion into the Knee-Joint

**Published:** 1907-11-16

**Authors:** William Bennett

**Affiliations:** A Post-graduate Lecture delivered at the London School of Clinical Medicine (Seamen's Hospital).


### PAINLESS EFFUSION INTO THE KNEE-JOINT.

By SIR WILLIAM BENNETT, K.C.V.O., F.R.C.S.

A Post-graduate Lecture delivered at the London School of Clinical Medicine (Seamen's Hospital).

It not uncommonly happens that a patient is led to seek advice by the accidental discovery of fluid in the knee-joint—"water on the knee," as it is popularly called. There is no doubt that painless effusion, of which the patient is quite unaware, occurs more commonly in the knee-joint or joints than is generally supposed; and its discovery, frequently after some slight injury, sometimes leads to errors in diagnosis, with unnecessary exaggeration of the importance of the condition by those who do not understand its exact significance.

The reason of the knee being more liable than other articulations to effusion probably lies in the fact that the leverage on it is greater than on most other joints; that it is at a certain mechanical disadvantage, especially in long-legged people; and that it is more vulnerable, inasmuch as its synovial apparatus is less protected by soft parts than that of any other major articulation in the body. It is true that at first sight the posterior aspect of the elbow appears to be very close under the skin, but as a matter of fact the part which is so nearly superficial is only the olecranon; the synovial membrane of the joint itself being in truth protected by the backward projection of that process. In the case of the knee, on the other hand, the synovial apparatus is singularly exposed to injury.

For clinical purposes painless effusion into the knee-joint may be divided into three classes: (1) Effusion, with or without local heat, occurring at any time of life; (2) effusion occurring in the young about the period of adolescence; and (3) effusion occurring in adults and old people. It is necessary to understand that the tension of the capsule of the knee-joint depends for the most part upon the tonicity of the muscles of the thigh and buttock; in other words, well-developed muscles there generally mean a high degree of tension and tone in the capsule of the knee. On the other hand, weak or flabby muscles in the same parts are always associated with more or less flaccidity of the capsule of the joint, and, of course, also of the synovial membrane. Further, the rigidity of the knee in various subjects differs to a great degree. One individual may have a knee so well set up as to prevent, in the extended condition of the limb, any lateral movement perceptible under ordinary circumstances. In another, the position of the limb being the same, there may be such an amount of lateral mobility in the knee as to justify the term "wobbling" being applied to it. Now this looseness of the knee-joint may be important, or it may not, and it consists of two kinds; in the anatomical variety the looseness of the joint is merely a condition natural to the individual (such individuals being frequently called "double-jointed") and not always confined to the knee-joint, although it may be more marked in that articulation than in any other. In the pathological variety it is the outcome of some defect or disease in connection with the joint itself or the muscles which support it. It is necessary for clinical purposes to be able to determine whether the looseness of the joint is of the anatomical or of the pathological type, because in connection with effusions into the knee it will be found that in the anatomical form of loose joint effusions are frequently happening and are in a general way of little real consequence. On the other hand, effusions occurring in any joint which has become loose from pathological causes may be of very great importance. The anatomical looseness can as a rule be distinguished from the pathological by the fact that it is bilateral, the two joints being affected equally or nearly so. In the pathological kind it is usually found that one joint is loose, the other being in a more or less natural condition of tone and strength. The due appreciation of the importance of the wobbling knee is of great clinical moment, because the gravity of effusion of the painless kind, or of any other kind for the matter of that, into the joint is in inverse ratio to the amount of the laxity of the articulation; the looser the joint anatomically, the more liable it will be to passive effusion, and the less important will the effusion be.

It is a fact of some importance in relation to the matter under discussion, and one which seems to be less commonly known than would be expected, that in the majority of young subjects whilst growing, especially if they be of the long-legged, anæmic type, it will be found that after any great amount of exercise some effusion into the knees takes place, unless the joints happen to be singularly well set up and strong. In most cases in which the knee is loose some effusion of fluid commonly occurs after much exercise—a very important point to bear in mind, in consequence of the tendency to refer accumulations of fluid after, for an example, a very energetic game of football to imaginary injury, with the result that treatment of a totally wrong kind may be resorted to. Effusion of this kind as a rule disappears spontaneously within twenty-four hours of the cessation of the exercise which has led to its production. With regard to the detection of fluid in the knee-joint, especially of this kind, the following points must be borne in mind. First of all, it is quite impossible to be sure whether there is or is not fluid in the knee-joint unless the patient is examined in a standing position. There may be, especially in a wobbling knee, a considerable quantity of fluid which will be imperceptible to the ordinary methods of examination if the patient be in the horizontal position. On the assumption of the erect position the fluid sags down towards the anterior and front part of the joint, in the neighbourhood of the ligamentum patellæ, and becomes readily manifest. But this examination alone is not sufficient to prove whether fluid exists or not, because in certain conditions, especially in cases of tubercle and in some cases of long-continued chronic or subacute inflammation of a non-tuberculous kind, there will be found on either side of the ligamentum patellæ and behind it a certain amount of thickening, which, in the erect position of the patient, is of exactly the same shape and appearance as that produced by the sagging down of fluid from the inside of the joint; it is therefore necessary, if such an appearance is seen in a knee-joint when the patient is in the erect position, to examine the part when the limb is horizontal, for the obvious reason that if the swelling is due to fluid it will disappear in that position; whilst on the other hand, if it is due to chronic thickening, it will remain the same.

We now pass on to the consideration of the clinical aspects of this condition. The chronic effusion of the painless kind, which is discovered accidentally in the manner which has been previously referred to, is generally unassociated with any local increase in the temperature of the part, and it may then as a local manifestation be considered, in the absence of any marked constitutional condition, to be of little importance. On the other hand, if associated with increased local heat, which will be very slight—because if the increased heat were great there would certainly be pain, and that, of course, would put the case outside the type of those which we are at present considering—the condition assumes much greater importance, especially in relation to tubercle. The question of tubercle in passive effusions into the joints—we are speaking now more particularly of the knee—is a very important one, because there is no doubt that there is an inclination to consider tubercle as a cause of these chronic effusions much too frequently. It is often regarded as being a "fault on the right side" to incline to the diagnosis of tubercle in cases of this type, because it is said that even if the condition be not tubercle, the treatment which is adopted on the assumption that it is, will, if not necessary, at all events do no harm. But that is a fallacious supposition. The treatment of these chronic joints by the long-continued rest which is usually applied in the treatment of tubercle is distinctly harmful in the event of the disease being of a non-tuberculous type, because, apart from liability to bring about an unnecessary ankylosis, if these chronic conditions which are not tuberculous be treated as if they were, there is reason for believing that an impetus may be given to the onset of tuberculous change. The treatment of chronic effusion without heat should invariably be massage with exercise rather than rest or confinement in splints.

Although painless effusion in cases of loose knee may be, as I have said, in itself of small account, its constant repetition becomes a matter of importance for three principal reasons: (1) The tendency to crippling from progressive weakening of the limb; (2) the marked liability to acute developments after comparatively slight injuries; and (3), perhaps the most important of all, the frequency with which internal derangement of the knee-joint occurs in cases of this type.

It is therefore obviously essential that laxness of the joint, when present in any degree, and especially when associated with recurrent effusion, should somehow be corrected. The best and most scientific method of effecting this in the majority of cases is by means of skilful massage and exercise; but should this fail, or should circumstances make its employment impracticable, nothing remains but the use of an instrumental support, which, whilst strong and light, will allow of the normal range of movement in the joint without restriction. The manufacture of this requires not only a highly skilled mechanician, but also one who has some adequate knowledge of the functions of the joint concerned.

### Painless Effusion Occurring in Young People.

If a considerable number of young people at about the period of adolescence be examined, it will be found that a substantial percentage have more or less effusion in the knee-joints, sometimes in only one, but generally in both, which is entirely unsuspected, the effusion in nearly all cases being more marked on one side than on the other. In some cases the condition is discovered accidentally by the nurse or parents, very frequently when the child is taking a bath, or something of that kind, which naturally leads immediately to advice being sought; more often than not the effusion is attributed to some injury, although the injury may have been so slight as to have passed unnoticed at the time. With regard to girls, it will be invariably found that there is some disturbance in connection with the genital system; that either the catamenial epoch is imminent, that it has been imperfectly established, or, having been established, it has been suspended for a time, leaving the constitution in that peculiar condition of upset which occurs in these subjects. In such circumstances it appears that a cure is not effected by any available means until the catamenial function is fully established, or, having been established, until any irregularities in connection with it have been corrected. I have known cases in which effusion of this type having commenced during the early part of the adolescent period continued for many years, to the age of twenty or twenty-five, or even later, when the catamenial irregularity has persisted. And in such cases I have known the effusion, in consequence of its chronicity, attributed to tubercle, to gout, to rheumatism, and other causes; and treatment adopted which has not only failed to cure the condition, but has led to a general habit of invalidism, and in some cases the ruin of a life.

With regard to boys, it is difficult sometimes to see why such an effusion should occur. But the subjects affected are generally of a nervous, irritable, and anæmic type; in fact, very much the same type as in the female. And there appears to be no doubt that the condition is due in some way or another to the disturbance in the genital system which takes place at this period; but in boys, unlike girls, the effusion invariably passes off at the end of adolescence, unless a habit of self-abuse has been acquired or unless the subject becomes anæmic or debilitated from some other cause. The proper appreciation of these cases, and the manner in which they should be treated, are obviously serious matters. From a practical standpoint the treatment required is very little; the important matter is rather to know what to avoid. First of all, on no account should these patients be laid up with the view of resting the limbs. That is the worst thing that can be done, for the reason that rest leads to flaccidity of the muscles of the thigh and other parts, and directly tends to that looseness of the knee-joint to which I referred just now, which is so prone to be associated with more or less effusion into the articulation.

### Painless Effusion in Adults and Old People.

The cases in which this may occur are so numerous that the time at our disposal does not permit me to deal with them all. There are some, however, which are of so much importance that I want especially to call your attention to them. I have mentioned the subject of the "wobbling knee," and have pointed out how it tends to the occurrence of effusion in people who are perfectly healthy. I also said just now that it is a harmful thing to attribute to doubtful injury the effusion which sometimes occurs in joints of this kind as a result of great exertion. On the other hand, it will be equally harmful to ignore altogether the effects of injury of a joint containing fluid in consequence of defective tonicity. The difference between the effusion of no importance in a knee of this type and the effusion which may be grave depends entirely upon the existence of a local increase of heat. Should there be no increase of heat, you may treat the effusion lightly. On the other hand, should there be heat, until its disappearance the case should be treated with assiduous care.

It is well to bear in mind that a similar effusion frequently occurs during the climacteric time, especially when uterine disorders—such, for example, as fibroids—exist. Primarily painless, some discomfort may arise in connection with this type should increase of local heat occur. The cases are of importance because the key to treatment lies with the uterine trouble, whatever it may be, and a cure of the articular effusion can only be brought about by the rectification or spontaneous disappearance of the uterine disorder. In one case which came under my observation a passive effusion of this kind, which had been pursuing the ordinary painless course for a long time, suppurated, apparently in consequence of the septic condition of the uterine cavity. So far as the treatment is concerned enough has been said. Massage to prevent loss of tone in the joint is indicated, and the question of the necessity for rest must be determined by the uterine condition. These cases, like others of which I have spoken, are too frequently thought to be gout, and a treatment adopted which, under the circumstances, is likely to be harmful and sometimes entails great waste of money in uselessly seeking relief at foreign or other "water-cure" places.

The acute septic infection of joints associated with some forms of intestinal trouble we are now becoming pretty familiar with; but, so far as I know, no mention has been made of painless effusion in connection with certain cases of what seems to be merely chronic indigestion. These cases are more commonly, I think, attributed to gout than any other type of joint affection. But at all events, whether they are technically gout or not, they do not respond to the ordinary treatment of gout; and they certainly do not improve, or at all events recover completely, so long as the intestinal condition remains untreated and uncured. It is fair to assume that such effusions are the result of chronic infection (septic) from the intestinal tract. It is, therefore, of particular importance, supposing we are faced with an effusion of this kind in both knee-joints—the condition is generally bilateral, although it may not always be so—to ascertain whether symptoms of gastro-intestinal irritation are present. In such cases it will usually be found that little or no progress will be made towards recovery by the knee until the patient has been subjected to a thorough course of intestinal antiseptic treatment.

Painless effusion in anæmia is not uncommon, and is only worthy of special mention on the following grounds—(1) Increased local heat is generally present to a slight degree, and (2) the resulting laxity of the joint is liable to be relatively greater than in any other type of the affection. The former of these is responsible, I fancy, for the tendency to consider such cases tuberculous, especially when the effusion is unilateral, as is often the case; the latter is important as indicating the line of local treatment to be adopted—namely, measures to preserve as fully as possible the muscular strength of the limb.

### Painless Purulent Effusion into the Knee.

Very extraordinary painless effusions are sometimes met with. One of the most remarkable which I have seen was in the case of a medical man of middle age, who, having been previously perfectly well, discovered that one of his knees was swollen. He had been working hard at the time, and he took but little notice of it, and continued to conduct his business as usual. When I saw him the left knee was distended with fluid, it was quite painless, and was without heat. At first sight, after the history which he had given me, I thought it was in all probability a case of Charcot's disease, as there were certain peculiarities suggestive of it; but I could not corroborate this diagnosis by any of the symptoms commonly associated with that condition. It is true that I could get no knee-jerk on the side on which the effusion was, but that was evidently because the effusion was so extensive and the parts so tense that I could not apply the proper test to produce it. Well, I treated him for a time with massage, but without any effect; in fact, I exhausted the whole of my ordinary resources for a case of the kind, and still no good followed. I then determined to aspirate the joint, with a view to exerting pressure and other means of treatment. To my surprise, on emptying the joint with the aspirator the fluid proved to be pure pus, slightly coloured by blood. It must be allowed that this was a remarkable case, because the man was in perfect health in every way except for the condition of his knee; and this, greatly swollen as it was, produced nothing but a feeling of tiredness. Upon having the pus examined in the ordinary way it was reported to be absolutely sterile. What the explanation of a case of that kind is I do not know. My main reason for mentioning it is that it points a moral in the matter of treatment. According to all surgical tenets, I ought to have laid that joint freely open, washed it out, and drained it. It was a joint which was full of pus; but having had experience in former times of the way in which these cold collections of pus can sometimes be cured without such a radical method of treatment as free incision, I did not adopt the heroic method, but was content to do the best I could by emptying the joint with the aspirator, allowing the patient still to get about; and so far as the case went it answered remarkably well. This is worth bearing in mind, because it emphasises a point which I often lay stress upon—that it is not essential in all cases to follow slavishly a routine treatment, such, for instance, as laying open a joint full of pus from chronic causes. I have only seen one other case of painless purulent effusion like this into a joint. The circumstances were very much the same, and I drew off in that case also a quantity of perfectly sterile pus. The patient recovered with a sound knee.

### Conclusions.

A large experience of the conditions in which painless effusions occur leads me to the following conclusions:—

1. Painless effusion into the knee-joint is primarily the result of constitutional conditions or defective structural arrangement.

2. In the absence of increased local heat, injury need not be considered as a cause, excepting in the case of great looseness of the joint and of tall, growing youths, when excessive exercise may be followed by transient effusion.

3. Whilst the treatment in all cases of painless effusion without increased local heat should be directed locally to the avoidance of muscle-waste and rectification of laxness of the joint by massage and exercise, a permanent cure can only be attained by the intelligent management of the primary constitutional defect.

4. There is no reason for suspecting tubercle as a cause of painless effusion, no matter how long it may exist or how resistent it may be to treatment, unless there is persistent increase of local heat.

